# Global research trends in the application of artificial intelligence in oncology care: a bibliometric study

**DOI:** 10.3389/fonc.2024.1456144

**Published:** 2025-01-07

**Authors:** Mianmian Xu, Yafang Chen, Tianen Wu, Yuyan Chen, Wanling Zhuang, Yinhui Huang, Chuanzhen Chen

**Affiliations:** ^1^ Department of Urinary Surgery, Jinjiang Municipal Hospital, Quanzhou, China; ^2^ Department of Neurology, Second Affiliated Hospital of Fujian Medical University, Quanzhou, Fujian, China; ^3^ Department of Neurology, Jinjiang Municipal Hospital, Quanzhou, China; ^4^ Department of Nursing, Jinjiang Municipal Hospital, Quanzhou, China

**Keywords:** artificial intelligence, oncology nursing, cancer prevention, bibliometrics, VOSviewer, CiteSpace, hot topics

## Abstract

**Objective:**

To use bibliometric methods to analyze the prospects and development trends of artificial intelligence(AI) in oncology nursing from 1994 to 2024, providing guidance and reference for oncology nursing professionals and researchers.

**Methods:**

The core set of the Web of Science database was searched for articles from 1994 to 2024. The R package “Bibliometrix” was used to analyze the main bibliometric features, creating a three-domain chart to display relationships among institutions, countries, and keywords. VOSviewer facilitated co-authorship analysis and its visualization was used for co- occurrence analysis. CiteSpace calculated citation bursts and keyword occurrences.

**Results:**

A total of 517 articles were retrieved, representing 80 countries/regions. The United States had the highest number of publications, with 188 articles (36.4%), followed by China with 79 articles (15.3%). The top 10 institutions in terms of publication output were all U.S.-based universities or cancer research institutes, with Harvard University ranking first. Prominent research teams, such as those led by Repici, Aerts, and Almangush, have made significant contributions to studies on AI in tumor risk factor identification and symptom management. In recent years, the keywords with the highest burst strength were “model” and “human papillomavirus.” The most studied tumor type was breast cancer. While Cancers published the highest number of articles, journals such as CA: A Cancer Journal for Clinicians and PLOS ONE had higher impact and citation rates.

**Conclusion:**

By analyzing the volume of AI literature in oncology nursing, combined with the statistical analysis of institutions, core authors, journals, and keywords, the research hotspots and trends in the application of AI in oncology nursing over the past 30 years are revealed. AI in oncology nursing is entering a stage of rapid development, providing valuable reference for scholars and professionals in the field.

## 1 Background

As society continues to develop, the incidence of tumors is rising annually, making them a significant threat to global public health. According to data published by the International Agency for Research on Cancer (IARC) (1), approximately 20 million new cancer cases were diagnosed globally in 2022, with 9.7 million resulting in death. By 2050, the number of cancer cases is projected to increase to 35 million. Oncology nursing involves oncology nurses working collaboratively to enhance the survival and quality of life of cancer patients by promoting healthy lifestyles, facilitating early detection, managing symptoms and side effects throughout the disease trajectory, optimizing quality of life, and addressing the unique needs of oncology patients (2).AI (AI) utilizes machine learning (ML), deep learning (DL), and other algorithms to simulate, extend, and enhance human brain functions, enabling computers to perform independent and autonomous intelligent activities (3). With advancements in chip storage, big data processing, and wireless communication technologies, AI research now focuses on machine learning, deep learning, clinical decision support systems (CDSS), and expert systems(ES) (4, 5). AI in oncology nursing can reduce nurses’ workload, enhance their capabilities, and meet various unmet patient care needs. As scientific studies increasingly focus on AI in oncology nursing, identifying research hotspots and trends becomes crucial for scholars and practitioners. Bibliometrics uses statistical data to analyze publications, describing or showing relationships between works. In 1987, Pritchard defined bibliometrics as “the application of mathematical and statistical methods to analyze textual information, revealing its nature and trends in disciplinary development” (6). Recently, bibliometrics has been widely used to study the characteristics of academic publications, including influential countries, journals, institutions, authors, publications, references, and keywords (7). Bibliometrics employs various tools to create graphs and charts that visualize collaborations among countries, institutions, and authors (8). It allows researchers to quickly stay updated on developments and frontiers in a specific research area. The integration of AI into oncology nursing has gained significant attention in recent academic discussions. Despite growing interest, a comprehensive bibliometric analysis of research trends in this area is still lacking. This paper aims to fill this gap by creating a detailed knowledge graph on AI applications in oncology nursing, offering valuable insights for future research and practice.

## 2 Methods

### 2.1 Data sources and search strategies

Web of Science (WoS), one of the most widely accessed academic databases, includes over 12,000 high-quality journals and comprehensive citation records (9). Thus, WoS was selected as the target database. The search strategy was: [TS(Topic Search) = (“tumor nursing” OR “cancer care” OR “tumor care” OR “oncological nursing” OR “cancer nursing” OR “oncology nursing” OR “oncologic nursing” OR “cancer management” OR “cancer prevention”) AND (“AI” OR “artificial intelligence” OR “artificial intelligent” OR “intelligence” OR “artificial intellectual” OR “deep learning” OR “machine learning” OR “pattern recognition” OR “neural network”)]. Inclusion criteria: (1) Published literature related to the search topic; (2) English-language articles. Exclusion criteria: (1) Popular science, research findings, patents, conferences, etc. ; (2) Duplicates, notifications, news articles; (3) Incomplete content. After searching and importing the literature into Zotero, two reviewers fully reviewed the texts, and 517 articles were finally included. Relevant articles were saved in plain. txt format for further study, including complete records and cited references.

### 2.2 Software tools and their functions

**Figure 1:**
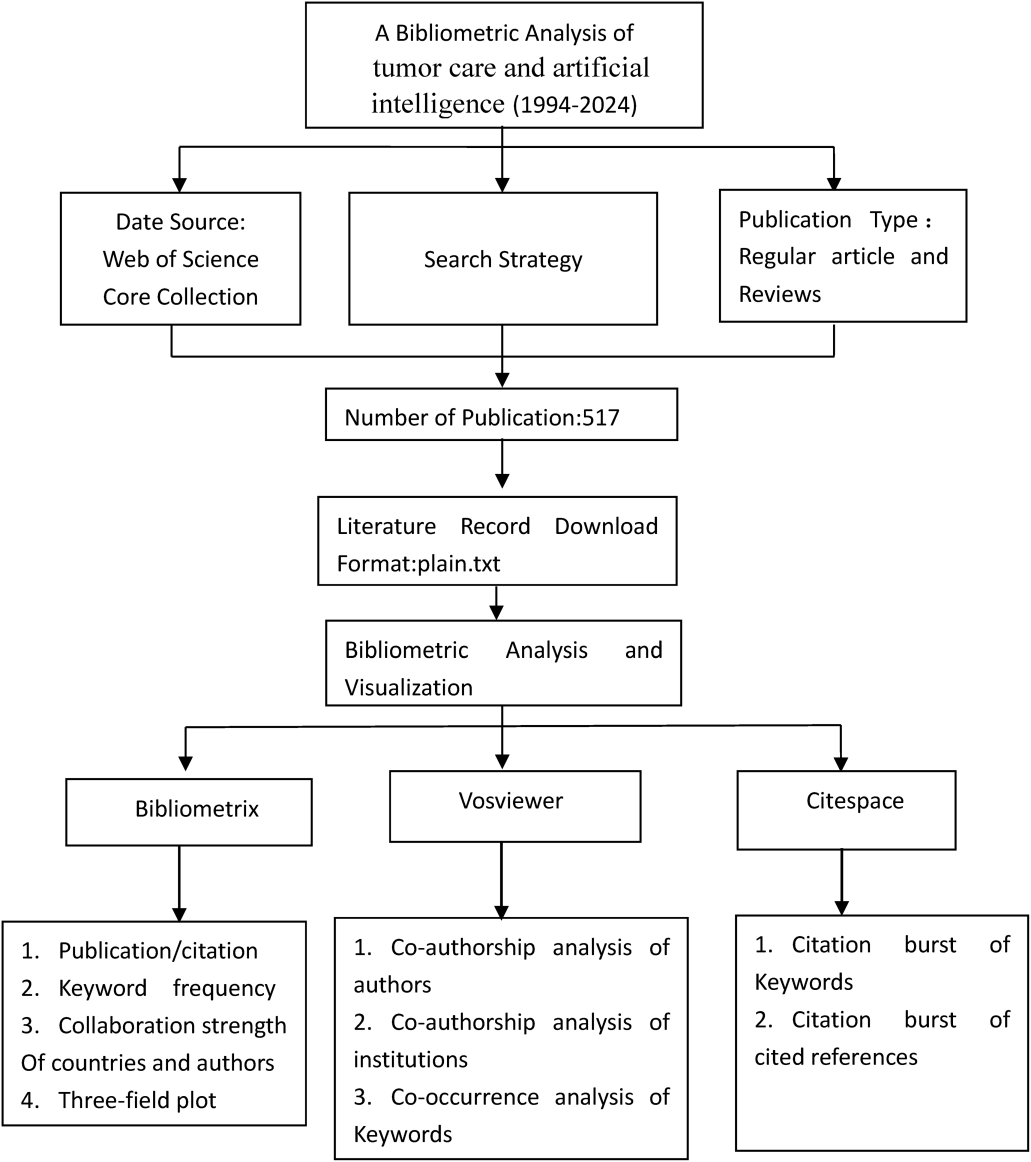
Workflow of the study Click here.

The tools employed for this bibliometric analysis encompass the Bibliometrix R package, VOSviewer, and CiteSpace. In this review, Bibliometrix version 4. 0. 0 is utilized to tally published papers and citations, calculate keyword frequency, assess collaboration strength between countries and authors, and generate a three-field diagram for keyword analysis. Temporal overlay features facilitate network visualization over time. In this paper, co-authorship analysis uncovers collaborations between authors and institutions, while co-occurrence analysis highlights associations between different keywords. Temporal overlay features facilitate network visualization over time. CiteSpace serves as a tool for citation analysis and visualization. In this study, CiteSpace identifies frequently cited papers and keywords over time with high citation frequency.**Figure 1** presents an overview of the bibliometric process.

## 3 Results

### 3.1 Annual publication analysis

**Figure 2:**
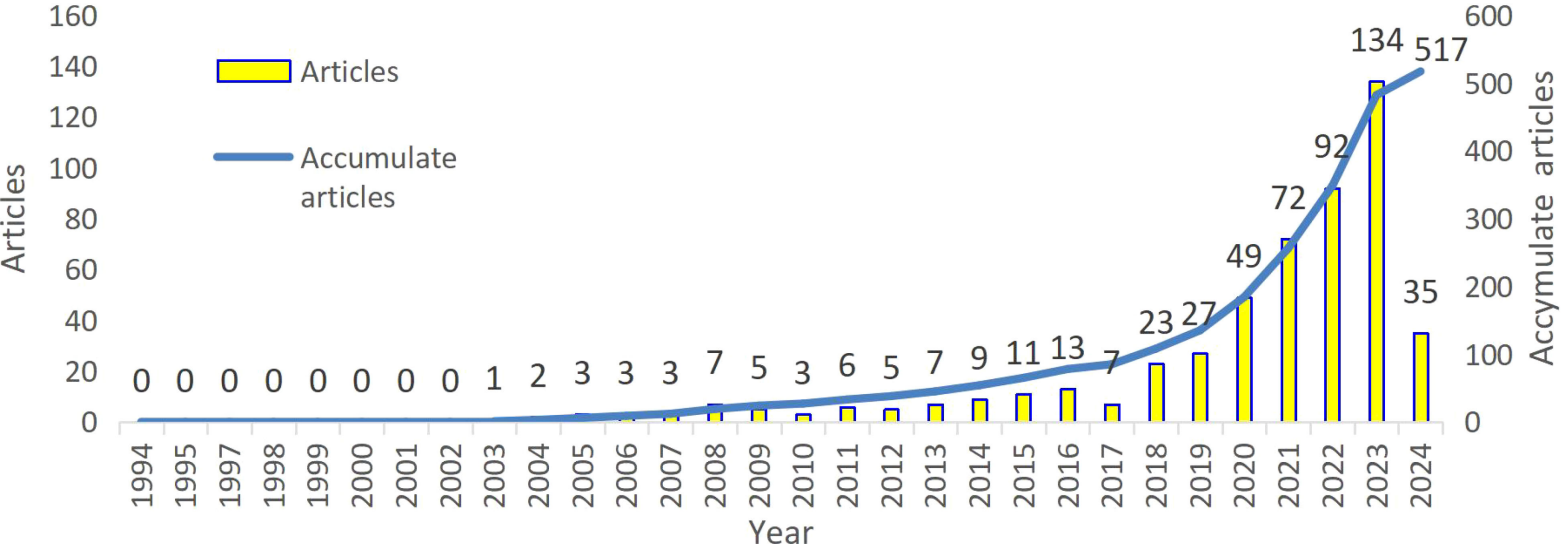
The annual number and the cumulative number of publications.

From March 31, 1994, to March 31, 2024, a total of 517 papers related to AI and oncology nursing were retrieved, after deduplication and screening, spanning a 30-year period.**Figure 2** shows the number of articles published each year and the cumulative number of articles. From 2003 to 2019, publications steadily increased from 1 article to 135, showing a gradual upward trend. In the last five years, from 2020 to 2024, the output grew rapidly, with a cumulative total of 517 publications by 2024. This highlights the growing attention of scholars to the application of AI in oncology nursing, and the rapid advancement of related research.

### 3.2 National publications and cooperation analysis

**Figure 3:**
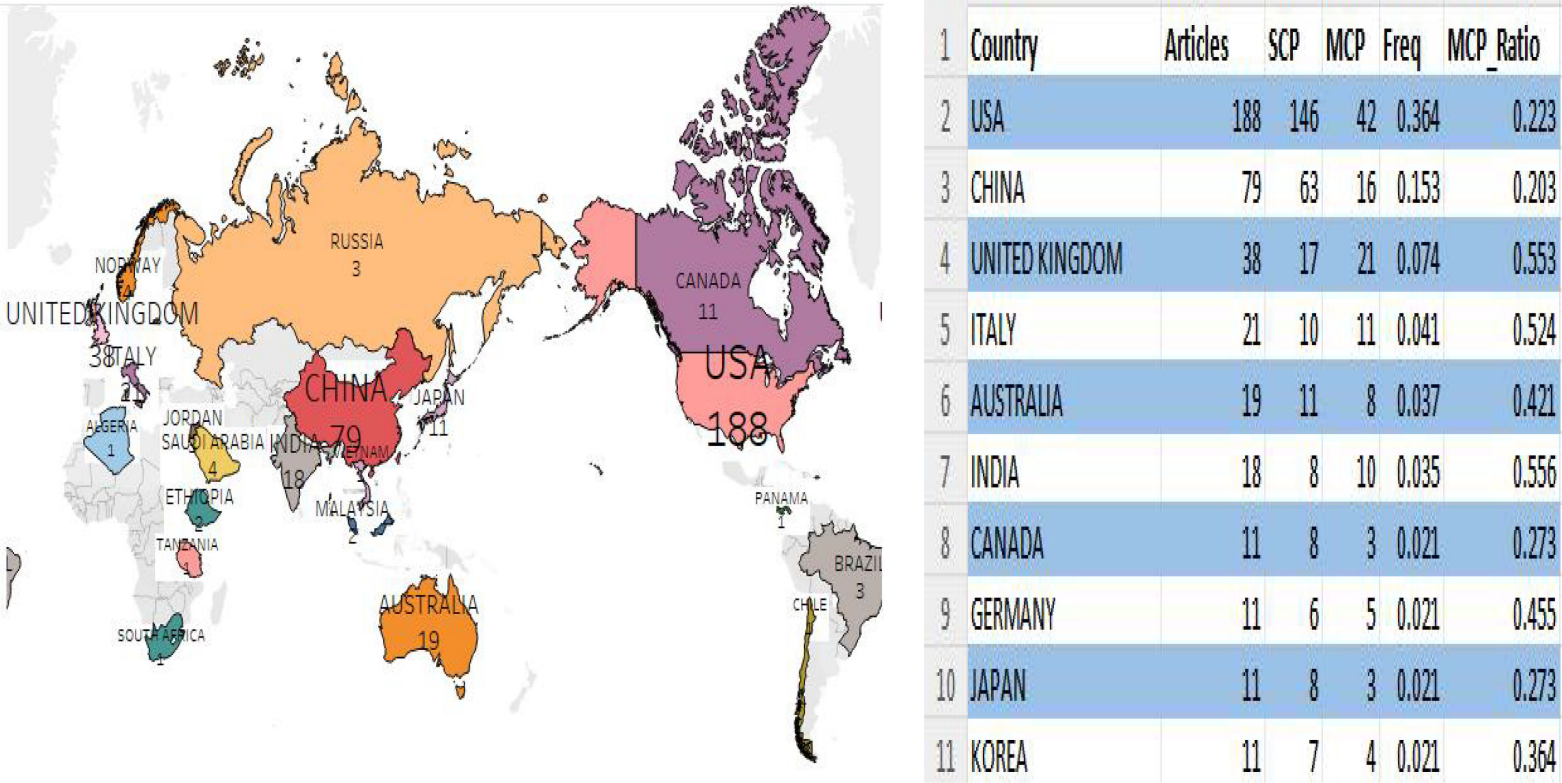
A map of country contribution based on the article output.

Analyzing national publication outputs shows contributions from 80 countries and territories to the literature in this field. As shown in **Figure 3**, the United States leads with 188 publications, accounting for 36. 4% of the total. China follows with 79 publications (15. 3%), the UK with 38 (7. 0%), and Italy with 21 (4. 1%). This distribution underscores the leading role of the United States in AI and oncology nursing research, with notable contributions from China, the UK, and Italy. Multi-Country Publications (MCP) indicate the number of publications by co-authors from various countries/regions. Although the US had the highest MCP (n=42), its MCP ratio (MCP/articles) was just 22. 3%. Further analysis of collaborations between countries/regions revealed the most frequent ones were between the U. S. and China and Italy (16), followed by the U. S. and the Netherlands (13), Germany (10), and England (10). Among the top 10 partnerships, all except the one between Spain and Italy involved the United States.

### 3.3 Analysis of institutional outputs and cooperation

**Figure 4:**
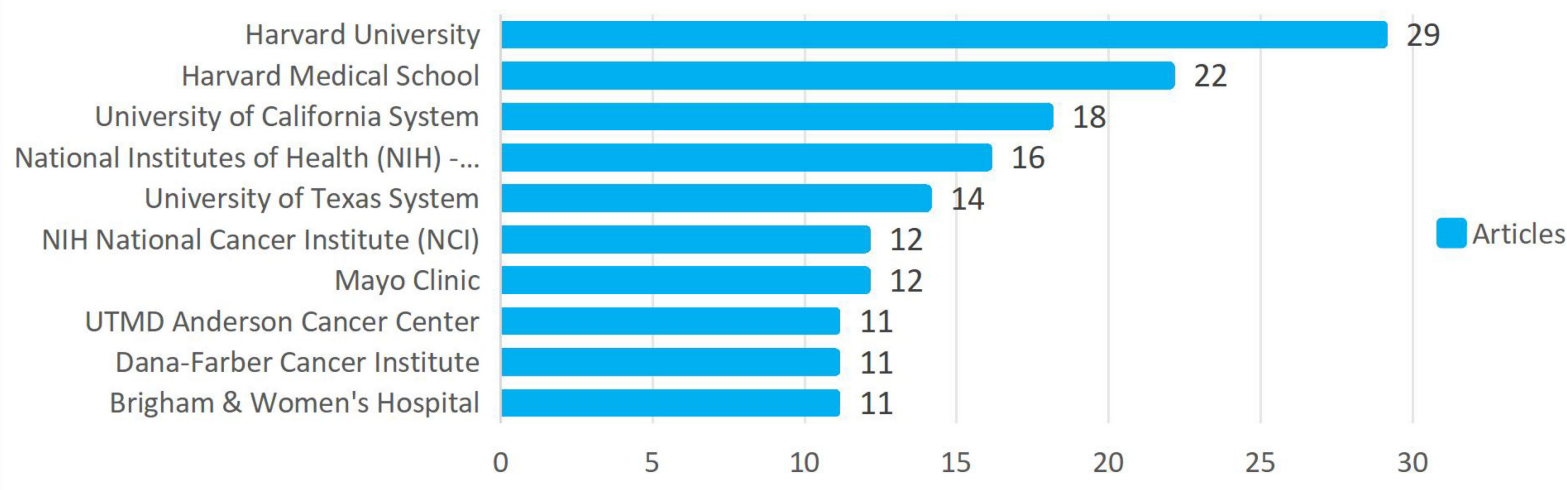
The top 10 institutions with the most publications.

The examination of journal article outputs and their impact shows that 358 institutions have engaged in AI and oncology care research.**Figure 4** highlights the top 10 institutions, all based in the United States, with Harvard University leading with 29 publications. This study also investigates collaboration among research institutions through co-authorship analysis, visualized in a clustering network. In this network, dot size represents the publication count per institution, while dot color signifies clusters based on collaboration intensity. Thirty institutions are divided into eight clusters, with the red cluster containing the most institutions, totaling ten. The clustering network with temporal overlap analysis reveals that dot colors indicate the average annual number of AI-related publications in oncology care. Early contributors in this field include institutions like Imperial College of Science and Technology and Unilever. Recently, Peking Union Medical College Hospital and the Chinese Academy of Sciences have been highly active in AI oncology research

### 3.4 Analyzing the output and impact of journal articles

Among all journals, 297 were involved in publishing the 517 articles retrieved in this study. Regarding journal impact, impact factor (10) and JCR (11) are effective indicators for assessing journal influence.**Table 1** lists the top 10 journals by article output along with their latest impact factors (IF). Both Cancers and Frontiers in Oncology, based in Switzerland, have similar influence, with Cancers ranking first, contributing 37 articles. Of the top 10 journals, two are categorized under Journal Citation Reports (JCR) Quartile 1 (Q1). Five publishers are based in the United States, two in the United Kingdom, and the remaining ones in Switzerland, Canada, and the Netherlands. Additionally, despite China’s significant contributions to the field, no Chinese journals have entered the top 10, indicating room for growth in establishing internationally influential journals.

**Table 1:** Top 10 journals with most articles about tumor care and artificial intelligence.

Rank	Journals	Articles	Country	IF	JCR
1	CANCERS	28	USA	5.2	Q2
2	FRONTIERS IN ONCOLOGY	19	Swiss	4.7	Q2
3	CANCER	8	USA	78.5	Q1
4	CUREUS JOURNAL OF MEDICAL SCIENCE	7	ENGLAND	1.5	Q3
5	JOURNAL OF MEDICAL INTERNET RESEARCH	7	Canada	7.4	Q1
6	PLOS ONE	7	USA	3.7	Q2
7	DIAGNOSTICS	6	Poland	3.6	Q3
8	JOURNAL OF CLINICAL MEDICINE	6	USA	3.9	Q2
9	CANCER MEDICINE	6	ENGLAND	4.0	Q2
10	JCO CLINICAL CANCER INFORMATICS	6	USA	3.2	Q2

### 3.5 Author impact and collaboration analysis

A total of 3414 authors contributed to studies on AI and oncology care.**Table 2** identifies Repici Alessandro as the most prolific author, with an H-index of 71 and 5 publications. He is followed by Aerts Hugo J. W. L. (H-index of 67), Almangush Alhadi (H-index of 26), and Alabi Rasheed Omobolaji (H-index of 12), each having 4 publications. Clustering network analysis revealed the collaborative relationships among these researchers. The size of the dots in the network represents the number of publications by each author, while the colors indicate different clusters based on collaboration strength. A total of 61 authors were grouped into 4 clusters, which were dispersed without forming a large, unified cluster. There was no collaboration between these different groups. A time-overlap plot of co-authorship analysis for 30 researchers highlighted Wenya Linda Bi MD and Luengo-Fernandez as active contributors to AI and oncology care research.

**Table 2:** Top 10 authors with the most articles about tumor care and artificial intelligence.

Rank	Authors	Articles	H- index	Institution and Country
1	Repici, Alessandro	5	71	Humanitas University, Italy
2	Aerts, Hugo J W L	4	67	Brigham and Women’s Hospital, USA
3	Almangush, Alhadi	4	26	Haartman Institute, Finland
4	Alabi, Rasheed Omobolaji	4	12	University of Helsinki, Finland University of Helsinki
5	Buchwald, Dedra	3	56	Washington State University, USA
6	Adikari, Achini	3	13	La Trobe University, Australia
7	Sharma, Prateek	3	63	University of Kansas, USA
8	Mori, Yuichi	3	31	University of Oslo, Norway
9	Hassan, Cesare	3	85	Humanitas University, Italy
10	Alahakoon, Damminda	3	9	La Trobe University, Australia

### 3.6 Most cited articles

The most-cited articles in a specific field reveal the impact of the research. An analysis of the top 10 most-cited articles shows that these publications appeared between 2017 and 2021, with 30% of them cited more than 15 times. The most-cited article, “Global Cancer Statistics 2020: GLOBOCAN Estimates of Incidence and Mortality Worldwide for 36 Cancers in 185 Countries,” was published in CA: A Cancer Journal for Clinicians in 2021 (12). This paper, using the latest data from the International Agency for Research on Cancer, highlights the global burden of cancer, with an estimated 19. 3 million new cases and nearly 10 million cancer deaths worldwide in 2020 (excluding non-melanoma skin cancer). Building advanced infrastructure for cancer prevention and care is crucial for global cancer control. The second most-cited article, “Artificial Intelligence in Cancer Imaging: Clinical Challenges and Applications,” published in CA: A Cancer Journal for Clinicians in 2019 (13), discusses the significant potential of AI in cancer imaging but notes challenges such as privacy, interpretability, and data quality. As technology and regulations evolve, AI is expected to play a key role in early detection, diagnosis, and personalized treatment of cancer.

### 3.7 Citation burst analysis

Citation burst analysis is a method provided by CiteSpace that identifies significant changes in references and keywords over a specific period. In citation burst analysis for references or keywords, burst strength indicates the intensity of attention and discussion a reference or keyword has attracted. A sudden citation burst reflects both the duration of citations and whether the reference has recently gained attention in the field. It also provides insight into research trends. The top 25 most-cited references are displayed in the citation burst analysis graph. A dark blue line represents the citation duration from 1994 to 2024, while the red line shows the period of the citation burst. The shortest burst period is two years. The strongest citation burst was for the article by Sung H et al. (12), titled “Global Cancer Statistics 2020: GLOBOCAN Estimates of Incidence and Mortality Worldwide for 36 Cancers in 185 Countries” (citation burst from 2022 to 2024, burst strength = 9. 17). This article highlights the global burden of cancer and the critical need to apply existing cancer control interventions and increase financial and technological support for prevention and treatment to reduce cancer mortality. With technological advancements, AI is increasingly demonstrating its unique advantages in cancer prevention and control. The second-strongest citation burst was for the article by Esteva A (14) titled “Dermatologist-level classification of skin cancer with deep neural networks” (citation burst strength = 5. 85). This paper applied a large dataset of clinical images to train deep neural networks, using pixels and disease labels as input, and demonstrated AI’s ability to classify skin cancer at a level comparable to dermatologists. Mobile devices equipped with deep neural networks could expand diagnostic coverage and reduce costs. Between 2023 and 2024, seven articles experienced consecutive citation bursts, with the highest burst strength of 3. 52 for the reference “High-performance medicine: the convergence of human and artificial intelligence” (15). The second most prominent was “Artificial Intelligence in Cancer Research and Precision Medicine” (16).

### 3.8 Keyword co-occurrence analysis

**Figure 5:**
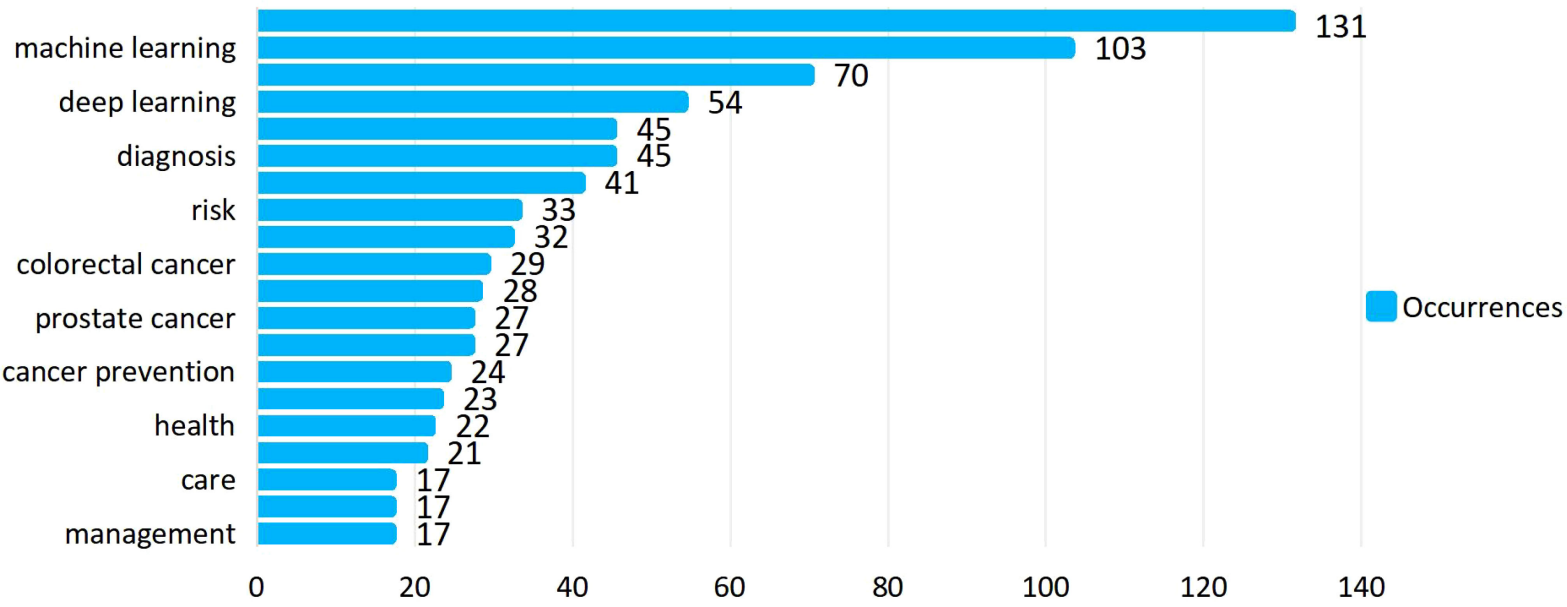
The top 10 most used keywords.

**Figure 6:**
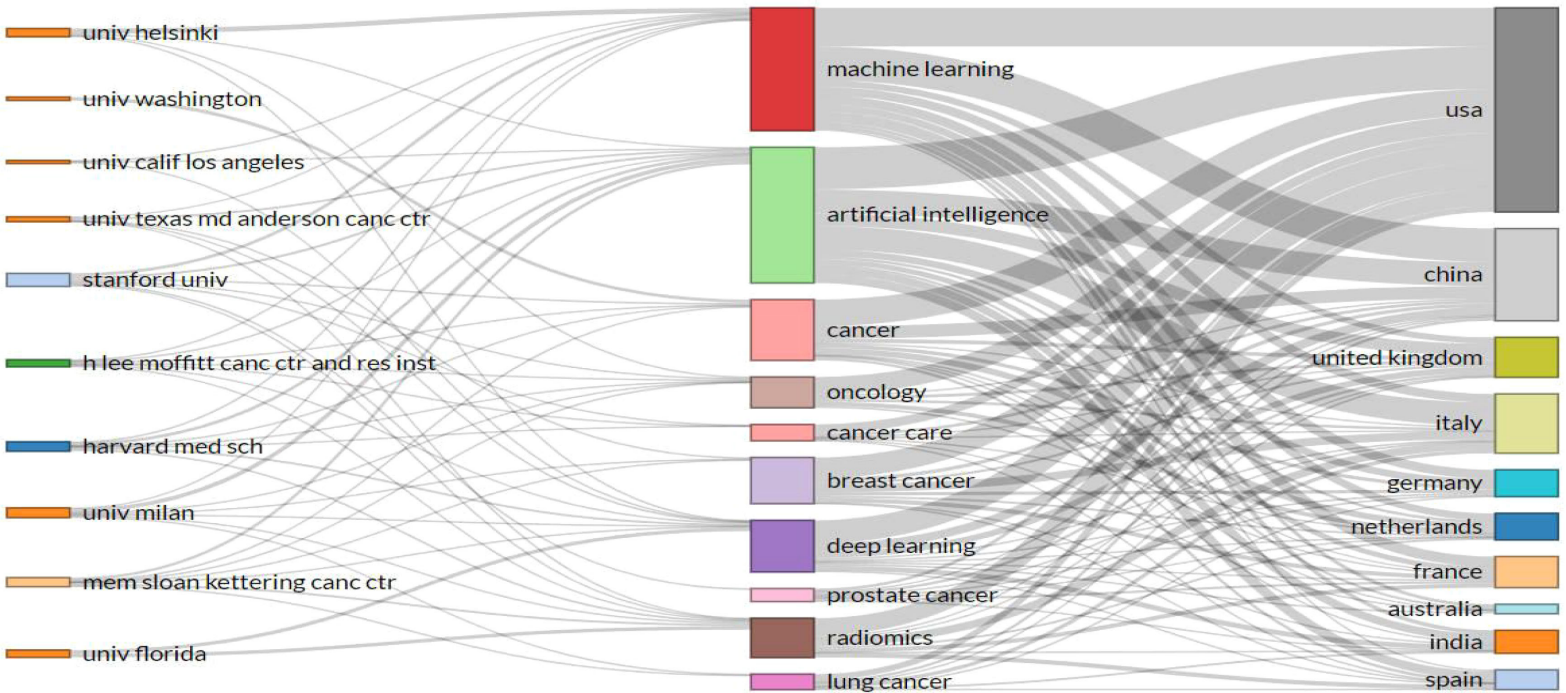
Three-field plot of the keywords plus analysis on tumor care and artificial intelligence (Left field: institutions; Middle field: keywords; Right field: countries).

**Figure 7:**
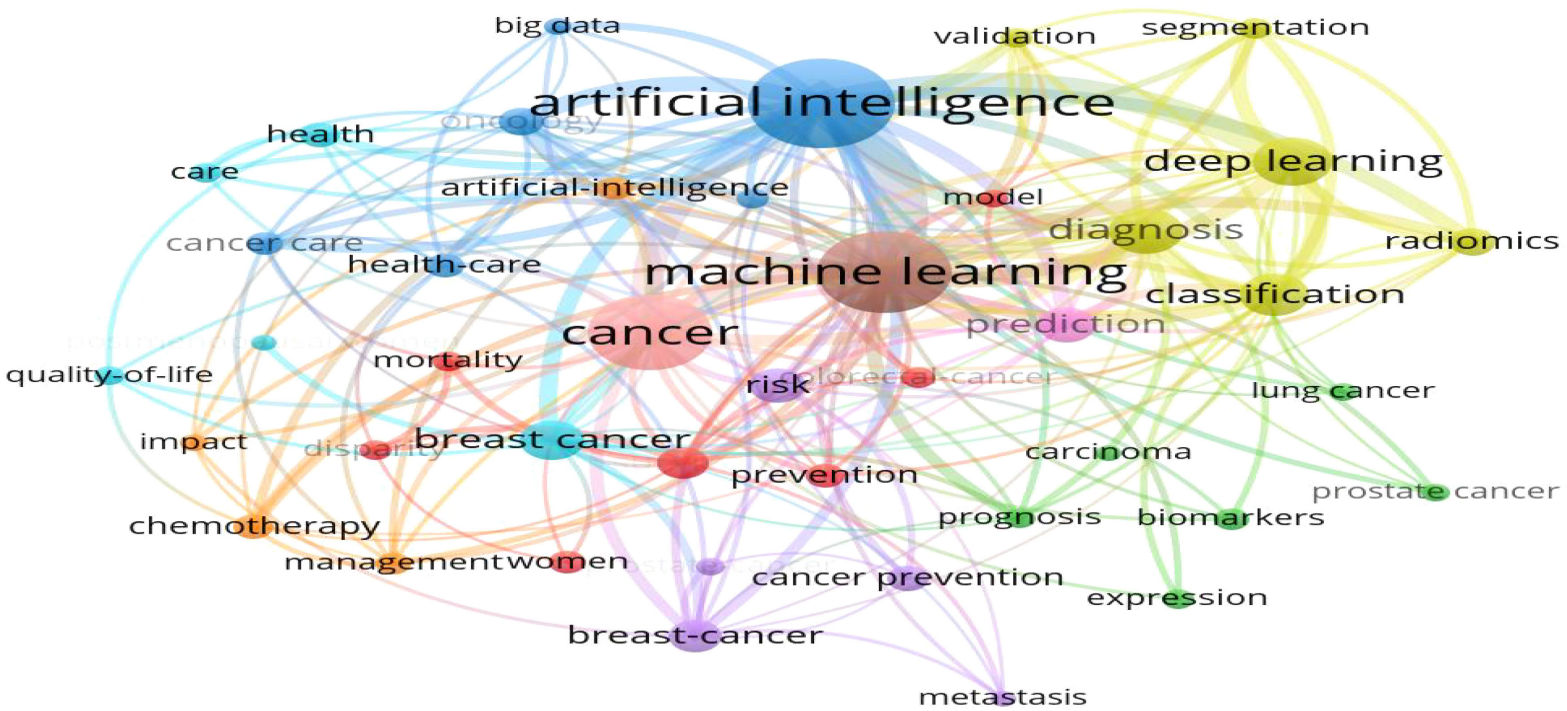
Keyword co-occurrence network (VOSviewew).

**Figure 8:**
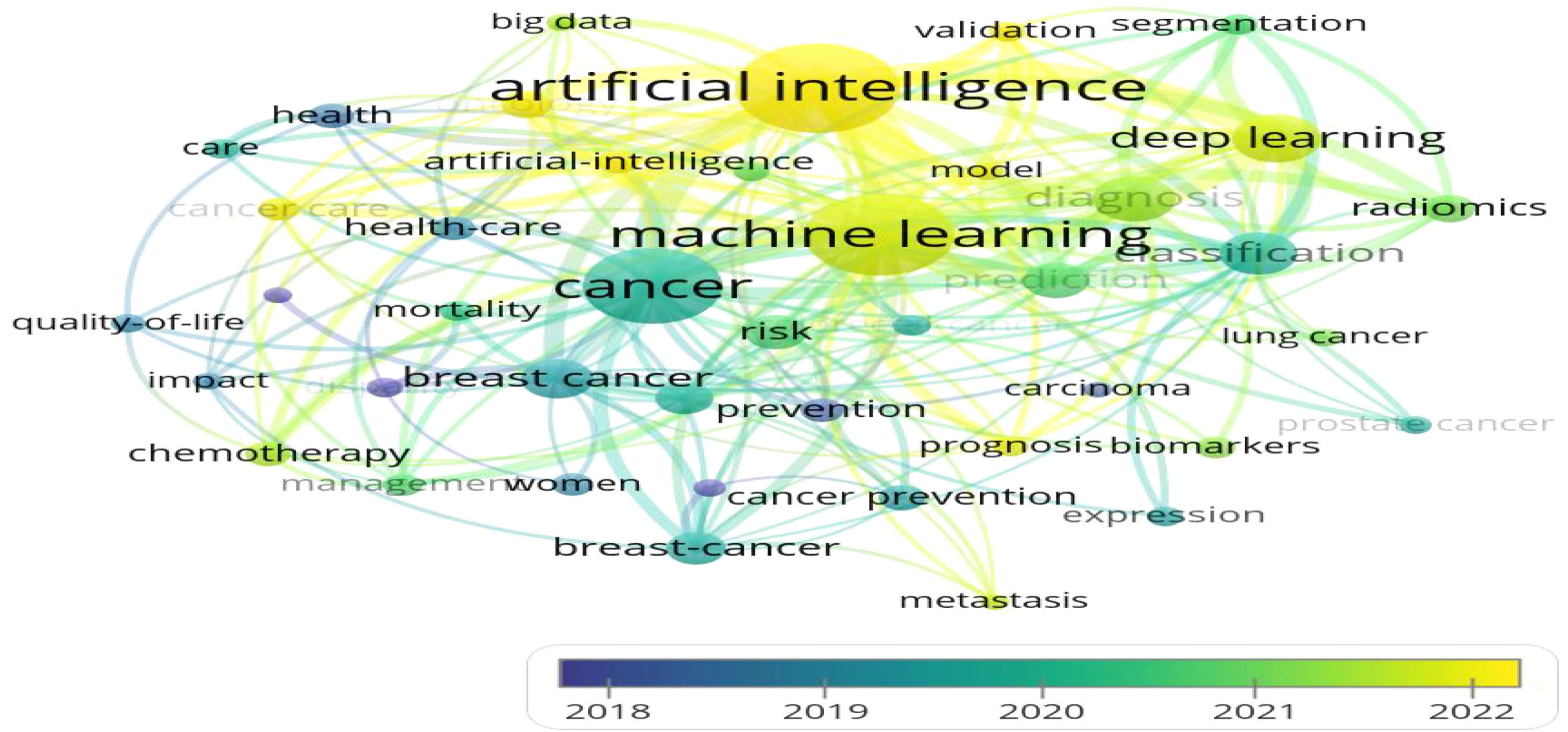
Keyword co-occurrence plus time-overlapping network (VOSviewew).

A total of 506 keywords were collected in this study.**Figure 5** displays the top 20 keywords ranked by frequency. The term “artificial intelligence” was the most frequent, appearing 131 times, followed by “machine learning” (n = 103) and “breast cancer” (n = 70). Among the top 20 keywords, cancer types such as “breast cancer” (n = 70), “colorectal cancer” (n = 29), “lung cancer” (n = 28), and “prostate cancer” (n = 27) were highly represented, all of which are associated with common cancer cases.**Figure 6** further illustrates the proportion of core topics for each institution and country, highlighting the connections and distributions between countries, institutions, and keywords related to AI and oncology. In general, nearly all institutions and countries contributed to the 11 themes represented by the keywords, though some differences were observed. For instance, the University of Helsinki showed a greater interest in “machine learning,” “deep learning,” and “artificial intelligence,” whereas the University of Washington was more involved in “cancer” research, with limited focus on AI applications in cancer prevention and treatment. Notably, Stanford University conducted research on “prostate cancer,” while the State University System of Florida concentrated more on “deep learning” and “radiomics. ” At the national level, the United States and China made significant contributions to these hot topics. However, China’s focus was more on AI than cancer prevention research, especially in keywords like “artificial intelligence,” “machine learning,” “breast cancer,” and “deep learning. ” In contrast, Germany exhibited lower interest in “oncology” and “cancer care,” and France showed less focus on “lung cancer. ” A co-occurrence analysis included 42 keywords.**Figure 7** shows the keyword network, where node size represents keyword frequency, node color indicates clusters, and line thickness reflects the strength of relationships between keywords. Keywords with closer associations were grouped into the same cluster. The 42 keywords were divided into 10 clusters. Cluster 1, colored red (containing seven keywords), focused on topics such as prognosis and prevention, including “disparity,” “model,” “mortality,” “prevention,” and specific cancer types like “colorectal cancer. ” Cluster 2, colored green (six keywords), concentrated on cancer biomarkers and expression, including keywords such as “biomarkers” and “expression,” as well as cancer types like “carcinoma,” “lung cancer,” and “prostate cancer. ” Cluster 3, in blue (six keywords), emphasized the application of modern technologies, including “artificial intelligence” and “big data,” as well as cancer care-related keywords such as “cancer care,” “health care,” “oncology,” and “radiotherapy. ” Yellow Cluster 4 focused on cancer diagnosis, with keywords like “radiomics,” “classification,” “diagnosis,” “segmentation,” “validation,” and “deep learning. ” Purple Cluster 5 primarily addressed cancer risks, including “cancer prevention,” “metastasis,” “risk,” and cancer types like “breast cancer” and “prostate cancer. ” Light blue Cluster 6 was concerned with cancer care, featuring keywords such as “care,” “health,” “quality of life,” and “breast cancer. ” The remaining two clusters, in orange and brown, contained other keywords like “artificial intelligence,” “machine learning,” “cancer,” and “prediction. ” **Figure 8** presents a temporal overlap analysis of these co-occurring keywords. The colors, ranging from dark blue to light blue and dark yellow, represent the average active years of the keywords. In the earlier stages of research, keywords such as “disparity,” “prevention,” and “carcinoma” were predominant. With technological advances, keywords like “artificial intelligence,” “machine learning,” “deep learning,” and “oncology” have gained prominence in recent years.

### 3.9 Keyword citation burst analysis

**Figure 9:**
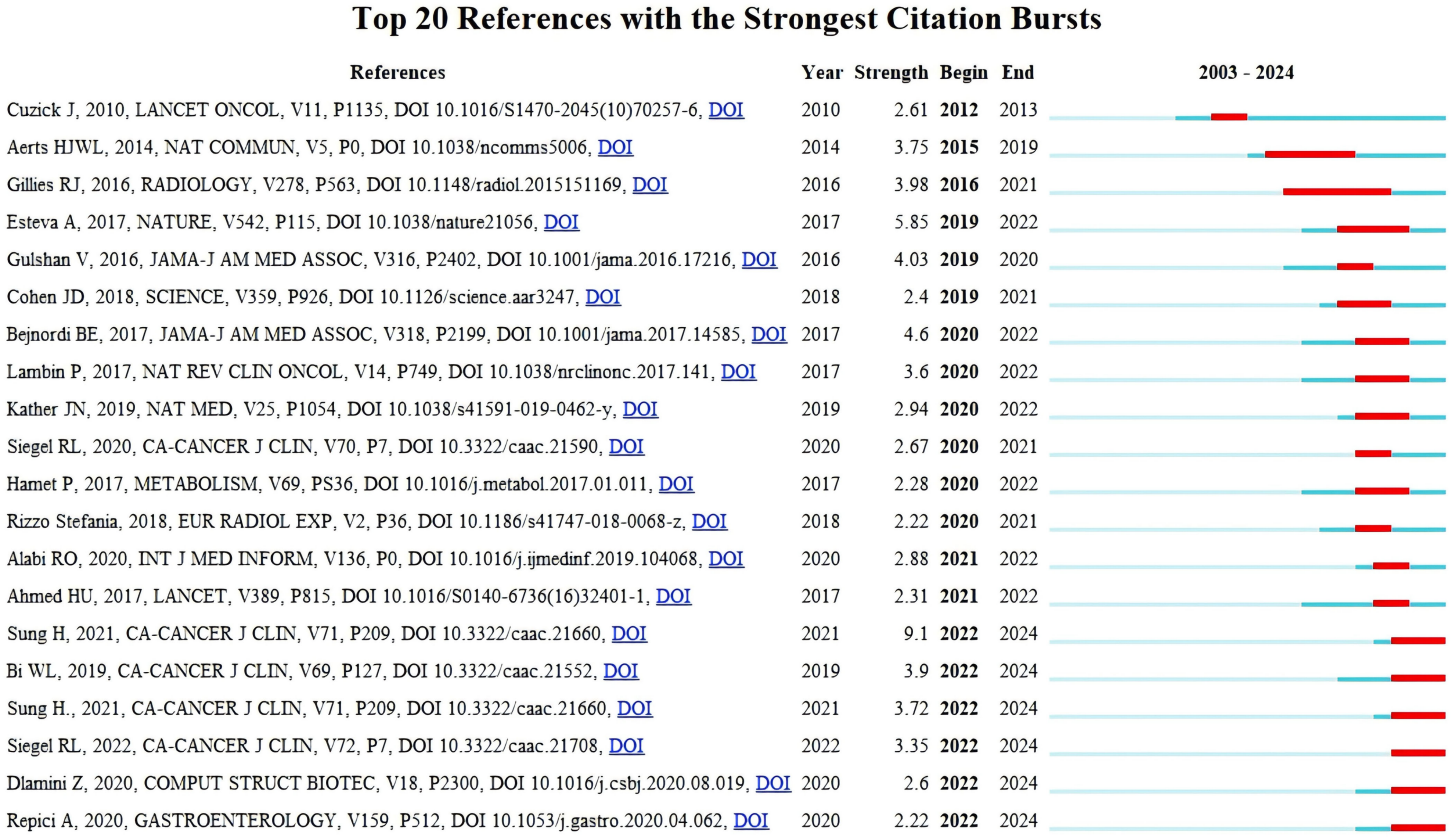
The top 25 keywords with robust citation bursts.

**Figure 9** highlights the top 25 keywords with the most significant citation bursts. “Alaska natives” (2004-2018, 14 years) and “artificial neural network” (2007-2017, 10 years) showed the longest citation bursts. Additionally, keywords such as “deep learning” (2019-2022), “trend” (2021-2022), “model” (2021-2024), and “human papillomavirus” (2022-2024) have gained considerable attention in recent years, indicating these keywords reflect popular and emerging research themes.

### 3.10 Analysis of different tumor types

**Table 3** highlights the most studied tumor types in the field of AI and oncology care. We counted the total number of articles for each tumor type. Tumor types with only one published paper, such as esophagogastric cancer and abdominal cancer, were categorized as “other. ” The top five tumor types were ranked based on the frequency of keywords in the top five articles. Breast cancer ranked first, appearing in 71 papers. Colorectal cancer, lung cancer, prostate cancer, cervical cancer, and bladder cancer followed, ranking from second to sixth.

**Table 3:** Top 13 interesting cancer types.

Rank	Tumor Type	Number
1	breast cancer	71
2	colorectal cancer	31
3	lung cancer	29
4	prostate cancer	27
5	cervical cancer	15
6	bladder cancer	7
7	gastrointestinal cancer	6
8	pancreatic cancer	4
9	esophageal cancers	4
10	ovarian cancer	3
11	tongue cancer	2
12	endometrial cancer	2
13	Other	2
	Total	203

## 4 Discussion

Over the past 20 years, research on the application of AI in oncology care has made significant progress. Using bibliometric tools, we objectively mapped the trends in this field, including the growth of annual publications, contributing countries and institutions, co-occurrence patterns of authors and keywords, and key research topics and hotspots. This study only included English-language articles from the Web of Science (WOS) database, which may have excluded some valuable research. However, given the broad coverage of WOS, our findings appropriately reflect the primary trends and focal points in this area. Based on the categorization of keywords, we highlighted the major research hotspots, recent advancements, challenges, and potential solutions in the intersection of AI and oncology care.

### 4.1 Advances in AI in oncology care

In 2003, Kirty S. Solanky et al. first applied biofluid nuclear magnetic resonance spectroscopy in a mouse model, discovering that tea polyphenols can influence the bioavailability and metabolic responses of living systems, with cancer prevention and anti-inflammatory effects. This marked a new chapter in the application of AI in cancer prevention and treatment (17). Based on the number of published articles, the growth trend can be divided into two phases: a slow growth phase and a rapid growth phase. From 2003 to 2019, the annual number of publications remained under 30, representing the slow growth phase. However, in 2020, a significant increase in annual publications related to AI and oncology care was observed. From 2020 to 2024, the field entered a phase of rapid growth, suggesting that research on AI in oncology care will likely remain active and robust in the coming years. The underlying cause of this trend may be the increasing role AI plays in transforming oncology care as AI technologies continue to advance. The growing attention and support from scholars in this area is one of the key drivers behind the recent surge in publications.

This study aims to analyze the scientific hotspots in the field of AI and oncology care by examining publications, references, and keywords. The cited papers reflect the core themes of a specific research area and help identify emerging research trends. Overall, the top ten most cited articles focus on several key areas: the growing global cancer burden driving AI development and the rapid expansion of AI in oncology-related disciplines. Among the most frequently cited publications, the American Cancer Society (18) projected that in 2022, there would be 1,918,030 new cancer cases and 609,360 cancer-related deaths in the United States, with approximately 350 daily deaths from lung cancer. Although advancements in early diagnosis, surgical techniques, and targeted therapies have reduced cancer mortality, the incidence of breast cancer and advanced prostate cancer has been exacerbated by disparities in race, socioeconomic status, geography, and unequal access to interventions. Reducing cancer mortality requires urgent implementation of existing cancer control measures and increased financial support for prevention and treatment technologies. With technological advancements, AI has demonstrated unique advantages in cancer prevention and treatment. In 2017, Esteva (14) trained deep neural networks using large clinical image datasets, achieving skin cancer classification performance comparable to that of specialists. Mobile devices equipped with deep neural networks have the potential to expand diagnostic accessibility and reduce costs. In 2018, Joshua D. Cohen (19) discovered that the CancerSEEK test, which detects eight common cancer types through blood tests, could identify early-stage cancers and determine the origin of the cancerous tissue. In 2019, Diego Ardila (20) applied a deep learning model to enhance the accuracy, consistency, and adoption of global lung cancer screening, creating opportunities to optimize the screening process through computer-assisted and automated approaches. The top 10 countries in this research area accounted for 78% of the total publications. The U. S. leads in both publications and international collaborations, reflecting its economic strength and substantial investments in healthcare. Although China ranks second in publications, it has fewer top institutions, indicating a need for enhanced research competitiveness through both domestic and international cooperation. With the highest total number of publications, nearly all of the top 20 organizations are from the United States. Despite China’s second rank in publications, only two of its institutions are in the top 20. England, ranking third in publications, has no institutions in the top 20. Two Chinese institutions, the Chinese Academy of Medical Sciences - Peking Union Medical College and the Chinese Academy of Sciences, have been particularly active in recent years. Enhancing research competitiveness through both domestic and international cooperation is crucial.

In the field of oncology care, Dean F. Sittig from the U. S. Health Research Center predicted in 2006 that cancer incidence rates for many types of cancer would significantly increase by 2015 (21). AI is expected to have the most impact on early detection, diagnosis, treatment, survival, and palliative care for patients, caregivers, and clinicians during cancer diagnosis and treatment. AI applications are likely to be first adopted by radiologists (20, 22) and pathologists (19) for early cancer detection and diagnosis, leveraging high-quality digital images and remote expert consultations. Clinicians, by reviewing patients’ electronic health records, can request local or remote cancer experts to assess current treatment plans, thereby improving diagnostic efficiency (23). Simultaneously, robotic surgery systems are being developed for remote surgical operations by surgeons (24, 25). Caregivers may also participate in post-discharge daily care through online platforms, helping patients and their families manage illnesses more actively and effectively (26, 27). In 2016, Dr. Richard Wagland from the University of Southampton’s School of Health Sciences developed and tested a machine learning-based text mining method for identifying specific free-text comments in large datasets, which facilitated the analysis of patient care experiences. This led to the creation of a model explaining the impact of care quality on health-related quality of life (HRQoL). The findings indicated that perceived care quality directly influences HRQoL (28). In 2019, Nathalie Reix (29) used machine learning to develop a new therapeutic decision tree incorporating uPA/PAI-1. The results suggested that uPA/PAI-1 alone should not be used as the sole indicator for chemotherapy in breast cancer care. Instead, a comprehensive assessment using additional biomarkers is needed, and further refinement of the decision tree is required to generate new chemotherapy indication protocols. At the beginning of 2020, Frank J. Penedo (30) published an article in Lancet Oncology, highlighting the growing value of e-health in providing patient-centered cancer care, marking a shift beyond traditional in-person care models to real-time, dynamic, and technology-assisted assessments and interventions. By improving communication between patients and providers, enhancing symptom and toxicity assessments, and optimizing patient engagement throughout the cancer care continuum, e-health has shown significant potential in delivering oncology care. As AI rapidly advances in oncology care, ethical issues have become more prominent. In 2024, Hantel A (31) conducted a cross-sectional survey of 204 oncology specialists regarding AI ethics in cancer treatment. The survey found that 84. 6% of U. S. oncologists believe oncologists should explain how AI models generate results, and 81. 4% agreed with using AI to assist in cancer treatment decisions. However, 47. 1% of oncologists felt that legal issues related to AI use in healthcare are the responsibility of physicians. Despite the majority of oncologists acknowledging the need to protect patients from biased AI, few felt confident in their ability to do so. These ethical concerns may hinder the effective application of AI in cancer treatment.

The most frequently used keywords are primarily related to “artificial intelligence,” including terms such as “machine learning,” “deep learning,” “big data,” “convolutional neural network,” “classification,” “diagnosis,” “prediction,” and “expression. ” A group of related keywords includes “cancer,” “breast cancer,” “colorectal cancer,” “lung cancer,” and “prostate cancer. ” Additionally, specific populations such as “women” and “postmenopausal women” are highlighted. However, temporal overlap analysis is not designed to identify the most commonly used keywords but rather to detect those that have been used more recently. Keywords such as “risk factors,” “system,” “deep learning,” “recurrence,” “trends,” “model,” and “human papillomavirus” represent current research hotspots. Regarding references, since 2022, six papers have experienced citation surges that have continued into 2024. Two significant studies—Sung H (12), which provided global cancer statistics, and Siegel RL (18), which reported new cancer cases and mortality in the U. S. —highlight the severity of the global cancer burden. In 2019, Dr. Wenya Linda Bi and colleagues (13) proposed that AI would enhance clinicians’ ability to interpret cancer imaging, gradually advancing AI technology toward clinical application and influencing the future direction of cancer treatment. In 2020, Italian gastroenterologist Alessandro Repici (32) evaluated the safety and effectiveness of a deep learning-based CADe system for detecting colorectal tumors, demonstrating that the system could reduce the miss rate of colorectal tumors during colonoscopy, showcasing AI’s advantages in endoscopic imaging. In the same year, another study explored the introduction of next-generation sequencing (NGS) platforms to address drug resistance and identify therapeutic targets, enhancing cancer diagnosis and prognosis prediction. This marked a new chapter in precision oncology and provided a foundation for cancer-specific follow-up care (33). The citation trends align with the keyword trends, emphasizing the role of AI in various medical fields, the development of cancer epidemiology, and the exploration of cancer risk factors through AI.

### 4.2 The application of AI in oncology care: challenges and prospects

Given the diverse patient population, various cancer types, diagnostic procedures, treatments, and disease trajectories, understanding how AI can be applied in oncology is crucial. This helps identify knowledge gaps that need to be addressed to improve patient care and professional practice. It also suggests necessary changes in cancer care education, clinical practice, research, and policy. In the near future, research on AI in oncology care may focus on several key areas: 1. Health Education and Awareness: Data analysis shows that increasing public health awareness has been proven to be a key factor in reducing cancer incidence (34). Researchers have developed various methods to enhance health awareness, including chatbots (35), game-based learning tools (36), animated videos (37), and social media (38), which play critical roles in promoting health-related knowledge. Since most exploratory studies have been conducted in developed countries (36), future research may expand to developing and underdeveloped regions to explore the applicability of AI in various cancer populations. 2. Symptom Management: Symptom management is a critical component of oncology care. Studies (39, 40) have shown that effective symptom management can lead to early detection of cancer progression and improved quality of care. With advancements in AI and the Internet of Things (IoT), remote and real-time symptom detection can assist healthcare providers in better managing symptoms. Beck (41) developed the “SymptomCare@Home” system, an AI-powered symptom monitoring and management tool. It provides daily symptom tracking, self-management guidance, and alerts for patients, assisting nurses with evaluation, education, support, and medication interventions to enhance symptom control outside the hospital. Smartphones equipped with location, gaming, and visual functions have been used to predict conditions such as depression (42), anxiety (43), pain (44), and cancer-related cognitive impairment (45), as well as assess patients’ quality of life (46). Remote symptom assessments can improve home care, enable early detection of complications, and enhance patients’ quality of life. 3. Care Coordination: Cancer patients often require multiple treatment modalities, involving coordination across various disciplines and hospitals. Care continuity is essential for patient outcomes (47, 48). To improve care coordination and symptom management, nurse navigators guide cancer patients through the care pathway from the initial imaging screening. AI can assist in directing, communicating, and supporting patients throughout the care process. It helps navigators predict emergencies, assess physical conditions, and provide consultations, enabling the automated planning of care pathways and allocation of appropriate healthcare units (49). AI-driven decisions are based on objective indicators such as pain, risk, and cost, allowing for rational resource allocation during care coordination (50, 51). In the future, AI-assisted systems will be refined to help nurse navigators improve care coordination, identify non-compliant care arrangements, and make necessary adjustments. 4. Drug Preparation and Injection: Traditional cancer drug preparation carries risks of accidental exposure through inhalation, ingestion, or skin absorption. With technological advancements, AI-powered drug preparation systems have made significant progress (52–54). Automated drug injection, particularly for oncology-related medications, is a growing area of interest. As research into biologics progresses, more cancer patients can receive treatment at home. AI enhances the safety of drug administration (55), provides intelligent analysis to increase patient engagement, and guides treatment decisions (56). AI-based disease management systems can track or predict patient outcomes, either independently or in clinical settings. The application of AI in drug development will further accelerate drug discovery and clinical trial design. AI models can screen large molecular datasets to predict potential anticancer drugs and design personalized drug combinations (55). 5. Decision Support: AI models can link histological features to clinical outcomes (22), offering opportunities to improve prognosis assessments. For example, using AI to classify cancers with a high likelihood of metastasis can help match patients with the most appropriate treatments, thereby avoiding overtreatment. Future research is likely to focus on precision oncology approaches related to AI (56). AI will play a key role in integrating multiple data types, such as genomics, imaging, pathology, and clinical data. Multimodal data integration is expected to provide a more comprehensive analysis of tumor characteristics, enhancing the precision and personalization of treatment (57). As patient-centered care continues to evolve, AI can facilitate proactive pain assessment and intervention, ensuring high-quality pain management for cancer patients. Intelligent systems (58, 59) can assist nurses in timely and convenient detection and alleviation of patient distress, enabling targeted care interventions for high-risk patients. 6. Clinical Practice Validation: As AI becomes more integrated into clinical oncology, cancer nurses will need additional training in machine learning and natural language processing. The lack of standardized health data in oncology hampers the testing, validation, certification, and auditing of AI algorithms and systems, limiting their generalizability and applicability. In the future, oncology nurses will need to incorporate AI tools into clinical practice only after predictive models have been clinically validated (60), further advancing the clinical application of AI in oncology care. In practice, oncology nurses must also emphasize the importance of addressing AI-related privacy concerns (61) and clinical accountability.

AI technologies, particularly machine learning (ML), deep learning (DL), and natural language processing (NLP), have demonstrated immense potential in handling large, complex datasets and automating analysis. ML is widely used in cancer diagnosis and prognosis prediction, especially for analyzing patient genomic data, medical images, and electronic health records (EHRs). DL techniques, particularly convolutional neural networks (CNNs), have made significant advances in medical imaging analysis, enabling automated tumor detection (62), optimization of radiotherapy planning (63), and integration of multimodal data (64). NLP shows great promise in automating the processing of medical text data and analyzing EHRs, with applications in oncology care, including automated EHR analysis (65) and literature reviews for knowledge extraction (66). However, key challenges remain for the broader application of AI in cancer care, particularly regarding explainability, generalizability, and integration into existing clinical workflows. The “black box” nature of AI limits clinicians’ ability to interpret its decisions. Developing interpretable models (e. g. , rule-based models or visualization tools) is essential to ensure the reliability of AI decisions and to build trust between clinicians and patients (67). AI systems must demonstrate strong generalizability across diverse patient populations to ensure their effectiveness across different racial, gender, and socioeconomic groups. However, many AI models are trained on data from specific populations, which can result in inaccurate diagnoses or inappropriate treatment recommendations. Future research should focus on ensuring data diversity, encompassing various races, genders, and pathological types, to reduce potential biases and improve the generalizability of models for all patient groups (68). Seamlessly integrating AI systems into existing clinical workflows presents a significant challenge (57). Clinicians are accustomed to their current work processes, and forcibly introducing new AI systems may increase workflow complexity and reduce efficiency. Therefore, AI tools must be designed to integrate with existing systems, such as electronic health records (EHRs), while maintaining ease of use and efficiency. Additionally, enhancing AI training for clinicians is essential so they can understand and apply AI-generated recommendations effectively. In cancer care, the integration of AI poses numerous challenges and ethical concerns, particularly related to data privacy, algorithmic bias, and the impact on the patient-provider relationship. AI systems rely on vast amounts of patient data, increasing the risk of privacy breaches. Patient data, if not properly anonymized, could be shared, leading to violations of privacy rights. For instance, AI systems based on EHRs may face risks of data leakage and misuse. Algorithmic bias can also negatively affect health outcomes for certain groups, especially those underrepresented in training data. This bias may result in misdiagnosis or improper treatment, particularly for patients of different races, genders, or socioeconomic backgrounds. Research shows that models trained on imbalanced data may fail to treat minority group patients fairly (68). The widespread use of AI could potentially weaken communication and trust between doctors and patients. As more decisions are made by AI systems, the role of doctors in providing personalized care may be diminished, which could affect patient experience and trust (69). Therefore, it is crucial to balance technological advancements with human-centered care when implementing AI.

This study has certain limitations. First, as a bibliometric analysis, the data collection and processing are highly dependent on software tools. Citespace uses time-slicing analysis to identify research evolution and emerging terms over different time periods. However, this strong reliance on methodology may lead to potential biases or insufficient coverage of research outcomes. Despite this, time-slicing analysis effectively illustrates dynamic changes within a field, offering a valuable perspective on evolutionary trends (70). VOSviewer, a distance-based visualization tool, may face limitations when analyzing smaller or incomplete datasets, and its dependence on database citations and keywords could result in inaccurate mapping. Nevertheless, VOSviewer provides structured visual representations of relationships between publications, offering an intuitive perspective for overall analysis (71). Bibliometrix, which leverages various R packages for quantitative visualization, requires higher programming skills from users, and its visualization tools are relatively basic. However, Bibliometrix offers flexible bibliometric capabilities, providing a highly scalable quantitative analysis approach and robust data mining abilities for comprehensive analysis (72). Secondly, this study only included English-language articles from the Web of Science (WOS) database, which may have resulted in the omission of some valuable research. However, given the broad coverage of WOS across most fields, this limitation is not expected to significantly impact the overall trend analysis. Thirdly, due to the citation lag, the influence of some recently published high-quality studies may be underestimated, which requires attention and updates in future research. Nevertheless, this study provides academic insights into the development trends, key topics, and emerging areas of AI in oncology care. In conclusion, the relationship between AI and oncology care is receiving increasing attention, with research in this area experiencing rapid growth. Future research should focus on several key directions: (1) the development and application of AI across medical fields, particularly in precision medicine; (2) personalized treatment plans; (3) clinical validation of AI prediction models; and (4) overcoming the challenges AI faces in oncology clinical practice. Through bibliometric analysis, this study systematically reveals the global research trends in AI applications in oncology care, providing valuable reference points for future research. As technology continues to advance, AI is expected to play an increasingly important role in promoting the intelligent and personalized development of healthcare services in oncology care.

## 5 Conclusions

The bibliometric analysis uncovers research hotspots and trends in AI applications within oncology nursing, indicating a rapidly evolving field with growing attention and support. Promising directions for future research include:1. The development and application of AI in various medical fields, especially in Health Science Awareness Dissemination and precision medicine. 2. Personalized treatment plans. 3. Clinical validation and practical application of AI predictive models. 4. Addressing challenges faced by AI in clinical oncology practice. In conclusion, this bibliometric analysis systematically reveals global research trends in the application of AI in oncology care, providing valuable insights and references for future research. With ongoing technological advancements, AI is expected to play an increasingly significant role in oncology care, driving the development of intelligent and personalized medical services.

## Data Availability

The original contributions presented in the study are included in the article/supplementary material. Further inquiries can be directed to the corresponding authors.

## References

[1] Bray F Laversanne M Sung H Ferlay J Siegel R Soerjomataram I .Global cancer statistics 2022: GLOBOCAN estimates of incidence and mortality worldwide for 36 cancers in 185 countries.CA Cancer J Clin. (2024) 74:229–63. doi: 10.3322/caac.21834 38572751

[2] The oncology nursing specialty. Oncol Nurs Forum. (2020) 47:125–6. doi: 10.1188/20.ONF.125-126 32078620

[3] Busnatu Ș Niculescu AG Bolocan A Petrescu G Păduraru D Năstasă I .Clinical applications of artificial intelligence—An updated overview.J Clin Med. (2022) 11:2265. doi: 10.3390/jcm11082265 35456357 PMC9031863

[4] Masters K .Artificial intelligence in medical education.Med Teach. (2019) 41:976–80. doi: 10.1080/0142159X.2019.1595557 31007106

[5] Haug CJ Drazen JM .Artificial intelligence and machine learning in clinical medicine. Reply.N Engl J Med. (2023) 388:2398–9. doi: 10.1056/NEJMra2302038 37342938

[6] RN B .Toward a definition of “bibliometrics. “.Scientometrics. (1987) 12:373–9.

[7] Ma D Yang B Guan B Song L Liu Q Fan Y .A bibliometric analysis of pyroptosis from 2001 to 2021.Front Immunol. (2021) 12:731933. doi: 10.3389/fimmu.2021.731933 34484243 PMC8416445

[8] Wang S Zhou H Zheng L Zhu W Zhu L Feng D .Global trends in research of macrophages associated with acute lung injury over the past 10 years: A bibliometric analysis.Front Immunol. (2021) 12:669539. doi: 10.3389/fimmu.2021.669539 34093568 PMC8173163

[9] Jiang S Liu Y Zheng H Zhang L Zhao H Sang X .Evolutionary patterns and research frontiers in neoadjuvant immunotherapy: A bibliometric analysis.Int J Surg. (2023) 109:2774–83. doi: 10.1097/JS9.0000000000000492 PMC10498839 37216225

[10] Woolston C .Impact factor abandoned by Dutch university in hiring and promotion decisions.Nature. (2021) 595:462. doi: 10.1038/d41586-021-01759-5 34172959

[11] Atallah ÁChecktae Puga M Amaral J .Web of Science journal citation report 2020: The Brazilian contribution to the "medicine, general & internal" category of the journal impact factor (JIF) ranking (SCI 2019).Sao Paulo Med J. (2020) 138:271–4. doi: 10.1590/1516-3180.2020.138419092020 PMC9673832 33111803

[12] Sung H Ferlay J Siegel RL Laversanne M Soerjomataram I Jemal A .Global cancer statistics 2020: GLOBOCAN estimates of incidence and mortality worldwide for 36 cancers in 185 countries.CA Cancer J Clin. (2021) 71:209–49. doi: 10.3322/caac.21660 33538338

[13] Bi WL Hosny A Schabath MB Giger ML Birkbak NJ Garraway LA .Artificial intelligence in cancer imaging: Clinical challenges and applications.CA Cancer J Clin. (2019) 69:127–57. doi: 10.3322/caac.21552 PMC6403009 30720861

[14] Esteva A Kuprel B Novoa RA Ko J Swetter SM Blau HM .Dermatologist-level classification of skin cancer with deep neural networks.Nature. (2017) 546:686. doi: 10.1038/nature22985 28658222

[15] Topol EJ .High-performance medicine: The convergence of human and artificial intelligence.Nat Med. (2019) 25:44–56. doi: 10.1038/s41591-018-0300-7 30617339

[16] Bhinder B Gilvary C Madhukar NS Elemento O .Artificial intelligence in cancer research and precision medicine.Cancer Discovery. (2021) 11:900–15. doi: 10.1158/2159-8290.CD-21-0090 PMC8034385 33811123

[17] Solanky KS Bailey NJ Holmes E Lindon J Davis A Mulder T .NMR-based metabonomic studies on the biochemical effects of epicatechin in the rat.J Agric Food Chem. (2003) 51:4139–45. doi: 10.1021/jf025677f 12822959

[18] Siegel RL Miller KD Fuchs HE Jemal A .Cancer statistics, 2022.CA Cancer J Clin. (2022) 72:7–33. doi: 10.3322/caac.21708 35020204

[19] Cohen JD Li L Wang Y Thoburn C Afsari B Danilova L .Detection and localization of surgically resectable cancers with a multi-analyte blood test.Science. (2018) 359:926–30. doi: 10.1126/science.aar3247 PMC6080308 29348365

[20] Ardila D Kiraly AP Bharadwaj S Choi B Reicher JJ Peng L .End-to-end lung cancer screening with three-dimensional deep learning on low-dose chest computed tomography.Nat Med. (2019) 25:1319. doi: 10.1038/s41591-019-0536-x 31253948

[21] Sittig DF .Potential impact of advanced clinical information technology on cancer care in 2015.Cancer Causes Control. (2006) 17:813–20. doi: 10.1007/s10552-006-0020-z 16783609

[22] Yao J Zhang Y Shen J Lei Z Xiong J Feng B .AI diagnosis of Bethesda category IV thyroid nodules.iScience. (2023) 26:108114. doi: 10.1016/j.isci.2023.108114 37867955 PMC10589877

[23] Branzan Albu A Laurendeau D Gurtner M Martel C .A web-based remote collaborative system for visualization and assessment of semi-automatic diagnosis of liver cancer from CT images.Stud Health Technol Inform. (2005) 111:75–8. 15718702

[24] Lee BR Png DJ Liew L Fabrizio M Li MK Jarrett JW .Laparoscopic telesurgery between the United States and Singapore.Ann Acad Med Singapore. (2000) 29:665–8. 11126706

[25] Melvin WS Needleman BJ Krause KR Ellison EC .Robotic resection of pancreatic neuroendocrine tumor.J Laparoendosc Adv Surg Tech A. (2003) 13:33–6. doi: 10.1089/109264203321235449 12676019

[26] Klemm P Bunnell D Cullen M Soneji R Gibbons P Holecek A .Online cancer support groups: A review of the research literature.Comput Inform Nurs. (2003) 21:136–42. doi: 10.1097/00024665-200305000-00010 12792194

[27] Gavrin JR .World Wide Web resources for cancer support groups.J Pain Palliat Care Pharmacother. (2005) 19:69–73. doi: 10.1080/J354v19n03_14 16219617

[28] Wagland R Recio-Saucedo A Simon M Bracher M Chable R Randhawa G .Development and testing of a text-mining approach to analyse patients' comments on their experiences of colorectal cancer care.BMJ Qual Saf. (2016) 25:604–14. doi: 10.1136/bmjqs-2015-004063 26512131

[29] Reix N Lodi M Jankowski S Delmas C Guillemin N Simon L .A novel machine learning-derived decision tree including uPA/PAI-1 for breast cancer care.Clin Chem Lab Med. (2019) 57:901–10. doi: 10.1515/cclm-2018-1065 30838840

[30] Penedo FJ Oswald LB Kronenfeld JP Garcia SF Cella D Yanez B .The increasing value of eHealth in the delivery of patient-centred cancer care.Lancet Oncol. (2020) 21:240–e251. doi: 10.1016/S1470-2045(20)30021-8 PMC7643123 32359500

[31] Hantel A Walsh TP Marron JM Kehl K Sharp R Van Allen E .Perspectives of oncologists on the ethical implications of using artificial intelligence for cancer care.JAMA Netw Open. (2024) 7:e244077. doi: 10.1001/jamanetworkopen.2024.4077 38546644 PMC10979310

[32] Repici A Badalamenti M Maselli R Correale L Radaelli F Rondonotti E .Efficacy of real-time computer-aided detection of colorectal neoplasia in a randomized trial.Gastroenterology. (2020) 159:512–520. e7. doi: 10.1053/j.gastro.2020.04.062 32371116

[33] Dlamini Z Francies FZ Hull R Marima R .Artificial intelligence (AI) and big data in cancer and precision oncology.Comput Struct Biotechnol J. (2020) 18:2300–11. doi: 10.1016/j.csbj.2020.08.019 PMC7490765 32994889

[34] Naz MSG Kariman N Ebadi A Ozgoli G Ghasemi V Fakari FR .Educational interventions for cervical cancer screening behavior of women: A systematic review.Asian Pac J Cancer Prev. (2018) 19:875–84. 10.22034/APJCP.2018.19.4.875 PMC6031778 29693331

[35] Daniel F Matera M Zaccaria V Dell’Orto A .Toward truly personal chatbots: On the development of custom conversational assistants. In: Proc. 1st Int. Workshop Softw. Eng. Cogn. Serv.ACM, New York, NY, USA (2018). p.31–6.

[36] Ruiz-López T Sen S Jakobsen E Tropé A Castle P Hansen BT .Fighthpv: Design and evaluation of a mobile game to raise awareness about human papillomavirus and nudge people to take action against cervical cancer.JMIR Serious Games. (2019) 7:e8540. doi: 10.2196/games.8540 30958271 PMC6475825

[37] Okpara CV Anselm AU Felix TO Omowale A Gever VC .The moderating role of colour in modelling the effectiveness of COVID-19 YouTube animated cartoons on the health behaviour of social media users in Nigeria.Health Promot Int. (2021) 36:1599–609. doi: 10.1093/heapro/daab001 PMC7989244 33729511

[38] Pervez MS Dutta P .Cancer survivorship in the digital era: Special reference to Facebook health groups.For Chem Rev. (2022), 1401–10.

[39] Denis F Koontz BF Letellier C .Application and benefits of web-mediated symptom reporting for patients undergoing immunotherapy: A clinical example.Case Rep Oncol. (2018) 11:763–8. doi: 10.1159/000494829 PMC6323363 30627090

[40] Kwekkeboom KL Wieben A Stevens J Tostrud L .Guideline-recommended symptom management strategies that cross over two or more cancer symptoms.Oncol Nurs Forum. (2020) 47:679–84. doi: 10.1188/20.ONF.498-511 32830800

[41] Beck SL Eaton LH Echeverria C Mooney K Wong B Whisenant M .Symptomcare@home: Developing an integrated symptom monitoring and management system for outpatients receiving chemotherapy.Comput Inform Nurs. (2017) 35:520–9. doi: 10.1097/CIN.0000000000000364 PMC5628091 28570285

[42] Saeb S Zhang M Karr CJ Schueller SM Corden ME Schmall JJ .Mobile phone sensor correlates of depressive symptom severity in daily-life behavior: An exploratory study.J Med Internet Res. (2015) 17:e175. doi: 10.2196/jmir.4273 26180009 PMC4526997

[43] Saeb S Lattie EG Kording KP Mohr D .Mobile phone detection of semantic location and its relationship to depression and anxiety.JMIR MHealth UHealth. (2017) 5:e112. doi: 10.2196/mhealth.7297 28798010 PMC5571235

[44] Chen L Ma X Zhu N Xue H Zeng H Chen H .Facial expression recognition with machine learning and assessment of distress in patients with cancer.Oncol Nurs Forum. (2021) 48:81–93. doi: 10.1188/21.ONF.81-93 33337433

[45] Small BJ Jim HSL Eisel SL .Cognitive performance of breast cancer survivors in daily life: Role of fatigue and depressed mood.Psycho-Oncology. (2019) 28:2174–80. doi: 10.1002/pon.v28.11 PMC6858929 31418499

[46] Granger CL Denehy L McDonald CF Irving L Clark RA Gough K .Physical activity measured using global positioning system tracking in nonsmall cell lung cancer: An observational study.Integr Cancer Ther. (2014) 13:482–92. doi: 10.1177/1534735414542484 25006040

[47] Gorin SS Haggstrom D Han PKJ Mooney K Rowland J Mandelblatt J .Cancer care coordination: A systematic review and meta-analysis of over 30 years of empirical studies.Ann Behav Med. (2017) 51:532–46. doi: 10.1007/s12160-017-9876-2 28685390

[48] Shulman LN Sheldon TA El Saghir NS Mukherjee RK .Multidisciplinary team-based care and the future of cancer treatment.Lancet Oncol. (2018) 19:e615–20.

[49] Moser EC Narayan G .Improving breast cancer care coordination and symptom management by using AI driven predictive toolkits.Breast (Edinb). (2020) 50:25–9. doi: 10.1016/j.breast.2019.12.006 PMC7375673 31978814

[50] Ivanics T Proctor E Chen Y Ali H Severson D Nasser H .Evaluation of a multidisciplinary team approach for generating survivorship care plan treatment summaries in patients with breast cancer.J Oncol Pract. (2019) 15:e467–74. doi: 10.1200/JOP.18.00509 30946641

[51] Walsh J Harrison JD Young JM Butow PN Solomon MJ White K .What are the current barriers to effective cancer care coordination? A Qual study. BMC Health Serv Res. (2010) 10:132. doi: 10.1186/1472-6963-10-132 PMC2891740 20482884

[52] Batson S Mitchell SA Lau D Wexler S Smith D Petrilli CM .Automated compounding technology and workflow solutions for the preparation of chemotherapy: A systematic review.Eur J Hosp Pharm. (2020) 27:330–6. doi: 10.1136/ejhpharm-2019-001948 PMC7856125 33097615

[53] Liu X Faes L Kale AU Wagner SK Fu DJ Bruynseels A .Artificial intelligence and deep learning in ophthalmology: A review and perspectives.Prog Retin Eye Res. (2019) 73:100767.

[54] Li Y Zhou Y Peng Y Chen Y Ai J Luo G .Application of artificial intelligence in pharmaceutical formulations: A review.Artif Intell Med. (2021) 117:102115. doi: 10.1016/j.artmed.2021.102115

[55] National Cancer Institute .NIH researchers develop AI tool with potential to more precisely match cancer drugs to patients.Nat Cancer. (2024).

[56] Sharpless NE Kerlavage AR .The potential of AI in cancer care and research.Biochim Biophys Acta Rev Cancer. (2021) 1876:188573. doi: 10.1016/j.bbcan.2021.188573 34052390

[57] Lotter W Hassett MJ Schultz N Kehl KL Van Allen EM Cerami E .Artificial intelligence in oncology: Current landscape, challenges, and future directions.Cancer Discovery. (2024) 14:711–26. doi: 10.1158/2159-8290.CD-23-1199 PMC11131133 38597966

[58] Decker VB Howard GS Holdread H Decker B Hamilton R .Piloting an automated distress management program in an oncology practice.Clin J Oncol Nurs. (2016) 20:E9–E15. doi: 10.1188/16.CJON.E9-E15 26800420

[59] Olling K Nyeng DW Wee L .Predicting acute odynophagia during lung cancer radiotherapy using observations derived from patient-centred nursing care.Tech Innov Patient Support Radiat Oncol. (2018) 5:16–20. doi: 10.1016/j.tipsro.2018.01.002 32095570 PMC7033763

[60] Van de Sande D Van Genderen ME Verhoef C Van Bommel J Gommers D Van Unen E .Predicting need for hospital-specific interventional care after surgery using electronic health record data.Surgery. (2021) 170:790–6. doi: 10.1016/j.surg.2021.05.005 34090676

[61] Ozair FF Jamshed N Sharma A Aggarwal P .Ethical issues in electronic health records: A general overview.Perspect Clin Res. (2015) 6:73–6. doi: 10.4103/2229-3485.153997 PMC4394583 25878950

[62] Litjens G Kooi T Bejnordi BE Setio A Ciompi F Ghafoorian M .A survey on deep learning in medical image analysis.Med Image Anal. (2017) 42:60–88. doi: 10.1016/j.media.2017.07.005 28778026

[63] Hosny A Parmar C Quackenbush J Schwartz LH Aerts HJWL .Artificial intelligence in radiology.Nat Rev Cancer. (2018) 18:500–10. doi: 10.1038/s41568-018-0016-5 PMC6268174 29777175

[64] Haenssle HA Fink C Schneiderbauer R Toberer F Buhl T Blum A .Man against machine: Diagnostic performance of a deep learning convolutional neural network for dermoscopic melanoma recognition in comparison to 58 dermatologists.Ann Oncol. (2018) 29:1836–42. doi: 10.1093/annonc/mdy166 29846502

[65] Rajkomar A Oren E Chen K .Scalable and accurate deep learning with electronic health records.NPJ Digit Med. (2018) 1:18. doi: 10.1038/s41746-018-0029-1 31304302 PMC6550175

[66] Wang Y Yu L Zhang Z Yan Y Zhang Y Li J .Deep learning-based natural language processing for EHR-based clinical decision support: A review.Artif Intell Med. (2019) 98:65–73.

[67] Ghassemi M Gusev A .Limiting bias in AI models for improved and equitable cancer care.Nat Rev Cancer. (2024) 24:197–209. doi: 10.1038/s41568-024-00739-x 39191902

[68] Topol E .The impact of bias in AI-driven healthcare: Challenges and considerations for equitable implementation.OxJournal. (2024) 10:85–102.

[69] Reller T .The ethics of medical AI and the physician-patient relationship.Cambridge Q Healthc Ethics. (2024) 33:123–40. 10.1017/S0963180119000847 31858938

[70] Chen C .CiteSpace: A practical guide for mapping scientific literature. Hauppauge, New York, USA: Nova Science Publishers (2016).

[71] Van Eck NJ Waltman L .Citation-based clustering of publications using CitNetExplorer and VOSviewer.Scientometrics. (2017) 111:1053–70. doi: 10.1007/s11192-017-2300-7 PMC5400793 28490825

[72] Aria M Cuccurullo C .Bibliometrix: An R-tool for comprehensive science mapping analysis.J Informetr. (2017) 11:959–75. doi: 10.1016/j.joi.2017.08.007

